# EXPLORING THE PERCEPTION OF PATIENTS WITH DEMENTIA ABOUT A SOCIAL ROBOT PARO IN A HOSPITAL SETTING

**DOI:** 10.1093/geroni/igz038.701

**Published:** 2019-11-08

**Authors:** Lillian Hung, mario Gregorio, Jim Mann, Neil Horne, Christine Wallsworth, Annette Berndt, Habib Chaudhury

**Affiliations:** 1 Simon Fraser University, Vancouver, British Columbia, Canada; 2 CEAN, Vancouver, British Columbia, Canada

## Abstract

PARO, a robotic pet, was designed to provide emotional and social support for older people with dementia. This project aims to explore the perception of persons with dementia about the role of PARO in a hospital setting. Video-ethnographic methods were applied. Patient and family partners were involved in the fieldwork of data collection and analysis. We conducted conversational interviews with ten patients with dementia staying in a geriatric unit and video observations. Thematic analysis yielded three substantive themes: (a) “it’s like a buddy”, the robot helps persons with dementia to uphold a sense of self in the world, (b) “it’s a conversation piece”, the baby seal facilitates social connection, and (c) “it’s all about love”, PARO transforms and humanizes the clinical setting. Our findings contribute to providing a better understanding of the direct perspectives of patients with dementia on the use of the social robot.

